# Multiple subcutaneous granulomas and severe rhinitis after intradermal deposition of epoxy: a case report

**DOI:** 10.1186/s12995-016-0120-y

**Published:** 2016-06-23

**Authors:** Steffen Roth, Anne Kristin Møller Fell

**Affiliations:** Department of Occupational and Environmental Medicine, Telemark Hospital, Ulefossveien 55, Skien, 3710 Norway; Department of Occupational and Environmental Medicine, University Hospital of North Norway, Tromsø, Norway

**Keywords:** Granuloma, Skin, Rhinitis, Work-related, Occupation, Epoxy

## Abstract

**Background:**

We present an unusual case of subcutaneous granulomas that also highlights the importance of assessing possible associations between exposure and symptoms early in the diagnostic approach to prevent further adverse health effects. Granulomas of the skin are seen in association with several diseases and after foreign body penetration of soft tissue, but have not been described after contact with epoxy. Epoxy resins are commonly used in paints and other protective coatings, including flooring materials.

**Case presentation:**

We report a case of granulomatous inflammation in a 58-year-old man after accidental intradermal deposition of unhardened epoxy. Multiple subcutaneous nodules were present on his right forearm, from hand to elbow, for a period of 6 months after the incident. Biopsies and histological analysis showed a granulomatous inflammation without necrosis. Microscopic analysis of the biopsies did not show mycobacterium tuberculosis, other bacteria, or fungal elements. Standard patch testing was negative. The nodules disappeared gradually, but intense pruritus remained. The patient returned to exposure and developed severe work related rhinitis.

**Conclusions:**

This case report describes an unusual case of multiple subcutaneous granulomas after a small injury with an epoxy-contaminated tool. Initially no association between the granulomas and exposure was established and the patient returned to work and epoxy exposure. He subsequently developed severe work related rhinitis. The case highlights the challenges of establishing an association between exposure and dermal reactions and that exposure should be reduced or avoided when sensitisation to allergens may have occurred.

## Background

Granulomas of the skin are seen in association with several diseases and after foreign body penetration of soft tissue, but have not been described following contact with epoxy. The granulomatous response is chronic inflammation characterized by focal collections of macrophages, epithelioid cells, and multinucleated giant cells, and may be accompanied by necrosis and fibrosis [1]. Granuloma formation is usually regarded as a means of defending the host from persistent exogenous or endogenous irritants that cannot be eliminated. Several diseases are characterized by the formation of granulomas, including rheumatoid arthritis, sarcoidosis, and leprosy [2]. Infections can result in the formation of granulomas, as in tuberculosis, schistosomiasis, histoplasmosis, and cryptococcosis. Granulomas are also associated with vasculitis [3] and can be found when a foreign body (such as glass, wood, or metal) penetrates the soft tissues of the body [4]. In 2012, a subset of severe asthma with granulomatous pathology was described for the first time [5].

Epoxy has a wide range of industrial applications, including metal coatings, use in electronic and electrical components, high-tension electrical insulators, fiber-reinforcing materials, and structural adhesives. Epoxy resins, also known as polyepoxides, are reactive prepolymers and polymers containing epoxide groups. Epoxy resins may self-react through catalytic homopolymerization, or combine with a wide range of coreactants including polyfunctional amines, acids, phenols, alcohols, and thiols. These coreactants are often referred to as hardeners or curatives. Reactions of polyepoxides with themselves or with polyfunctional hardeners form polymers that may have strong mechanical properties as well as high temperature and chemical resistance, and are thus widely used. The epoxy resin itself may be formed by a reaction between the substances bisphenol A diglycidyl and epichlorohydrin; these account for about 75 % of the epoxy resins used worldwide. Depending on the producer, the coreactants vary. Polyepoxides as highly reactive substances are known to be associated with work-related allergic contact dermatitis and occupational asthma [6]. A possible association between rhinitis and exposure to epoxy has been reported but is not well described in the literature [7]. We present an unusual case of subcutaneous granulomas that also highlights the importance of assessing possible associations between exposure and symptoms early in the diagnostic approach to prevent the development of further adverse health effects.

## Case presentation

A 58-year-old man presented with multiple lesions of the skin on his right forearm from the hand to the elbow. His medical history did not include any diseases associated with granulomatous inflammation or immunosuppression, and there was no family history of granulomatous disease. Prior to the patient’s current work, he had worked with epoxy resins for 4 years, repairing concrete constructions, in the period from 1988 to 1992. He had no exposure to epoxy in the period from 1992 to 1998 and had since then worked for 15 years as an industrial flooring specialist constructing epoxy floors. He had daily contact with polyepoxides, which he first would use as a coating on concrete floors. After hardening, he would use a primer over the first layer and add a final layer of epoxy compound. The patient blended the compounds together directly before he applied the mixture to the floor with a mason’s trowel. He reported sporadic use of gloves and a filter mask while working with epoxy compounds.

**Fig. 1:**
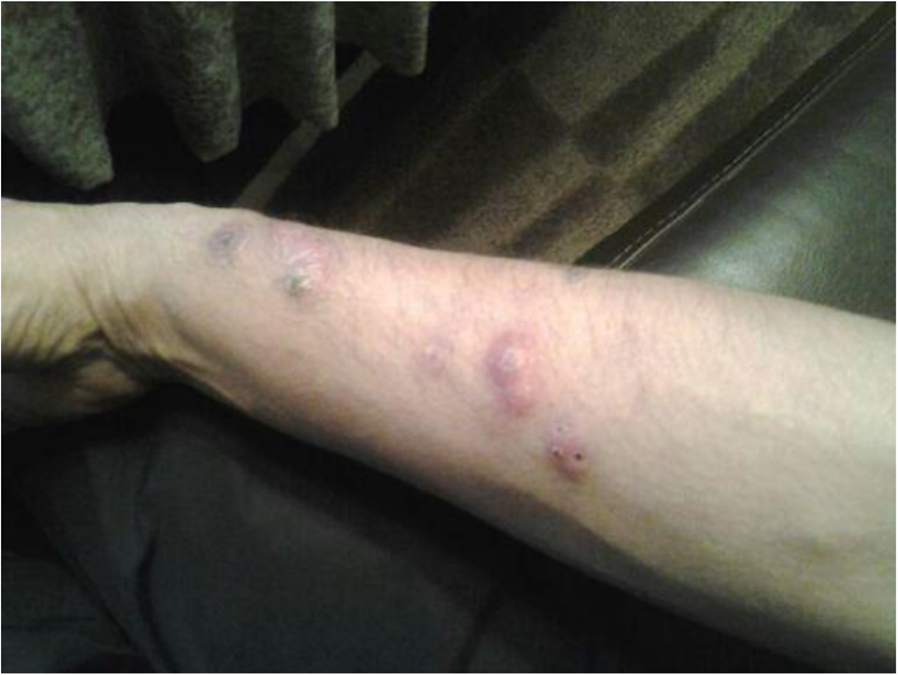
The patients right arm two weeks after the intradermal deposition of unhardend epoxy

In August 2013, he accidentally cut himself on the second digit of the right hand with the mason’s trowel contaminated with unhardened epoxy. During the following 10 days, he developed swelling with redness and pain of the digit, which spread to his palm. Because of the increasing symptoms, the patient contacted his general practitioner, 20 days after the accident. The level of C-reactive protein (CRP) was then < 5 mg/L. The wound was cleaned but the patient reported that some of the hardened compound remained in the depth of the wound. The patient was given dicloxacillin (500 mg × 4). The wound swabs did not show any growth of aerobic or anaerobic pathogens. In the following days, after another visit to the emergency ward because of an increase of the swelling, he was given erythromycin (250 mg × 2). At this point, the level of CRP was still < 5 mg/L. Thereafter, during the next weeks, the swelling and pain of his finger and palm disappeared. At the same time, multiple nodules (Fig. 1) appeared on the dorsal side of his right forearm. The nodules were surrounded by swelling and redness and the patient reported pruritus. He was referred to the internal medicine ward. CRP, erythrocyte sedimentation rate, leukocytes, and angiotensin-converting enzyme were within normal limits. Two biopsies were taken from the nodules, and the patient was given cetirizine hydrochloride (10 mg × 2) and prednisolon (20 mg × 1, two weeks treatment) with minor effect. The first biopsy showed granulomatous inflammation without necrosis/necrobiosis and no signs of vasculitis, while the second biopsy showed lymphohistocytic inflammation with mild eosinophilic involvement. Microscopic analysis of the biopsies did not show mycobacterium tuberculosis, other bacteria, or fungal elements (Periodic acid-Schiff and Ziehl-Neelsen stains) and the patient was released. The nodules increased in size during the subsequent weeks and varied between 1 and 3 cm in diameter. The patient was referred to a dermatologist for standard patch testing including epoxy, which was negative. The patient had no variation in his general well-being, but several more nodules appeared proximal of the first ones.

**Fig. 2:**
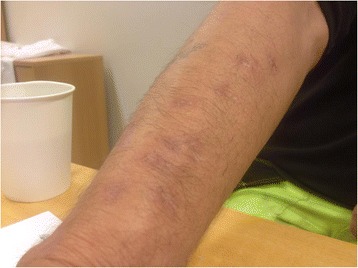
The patients arm six months after the intradermal deposition of epoxy

During the period of medical investigation, the patient did not work and had no further exposure. He reported that he manipulated the granulomas by squeezing, whereby they would disappear some days later. The granulomas did not spread proximal of his right elbow. All granulomas had disappeared six months after the day of intradermal deposition of epoxy resins (Fig. 2). However, pruritus in the previously affected area remained. The patient then returned to his work and epoxy exposure continued. Shortly thereafter, he developed rhinitis with symptoms limited to exposure at work and was referred to the department of occupational and environmental medicine. The patient reported that the rhinitis symptoms had increased during the past weeks and that frequent sneezing and a constantly runny nose affected his ability to work. The symptoms improved when he was absent from work, indicating that the patient had developed occupational rhinitis. The patients’ medical history did not include any information on former allergic reactions. Standard allergy testing (inhalation panel IP-6 and IP-7) did not reveal any allergies, but the patients total serum immunoglobulin-E (IgE) level increased (from 325 to 435 units) between two visits in our department. We wanted to send the patient to a centre specialized in specific inhalation challenge tests (SiC). However, because such testing requires several hours of traveling and the patient feared worsening of his rhinitis, he refused further testing.

## Discussion

Epoxy resins can cause both immediate and delayed allergic reactions, but immediate reactions are rare [8–10]. It is known also that components associated with epoxy resins such as acid anhydrides have potent direct irritant effects on the skin [7] and thus may induce irritant dermatitis. Our patient developed granulomas in a large area around the wound, thus embolization of material from the contaminated tool may be considered as a possible mechanism of the granulomas. Displacement of bisphenol A polycarbonate, a polymer commonly used in biomedical devices such as indwelling catheters and granulomas of the lung [11], has been described in one case report. It is also known that indwelling biomedical devices can disintegrate and embolize into lymphatic vessels. As an example, foreign body granulomas may occur locally after injection of silicone in cosmetic procedures [12].

In the present case, allergy to epoxy was suspected and standard patch testing performed. However, no allergy against epoxy was revealed and the patient returned to his workplace. His IgE level increased between two visits in our department. This may indicate an allergic response, but could not be directly attributed to the epoxy exposure because we had no evidence of a specific allergic reaction. Most commonly, epoxy exposures will result in irritant or allergic contact dermatitis that will not result in elevations of total IgE. Exposure to low molecule weight chemicals such as epoxy can, however, result in mixed immunological responses which may be IgE-mediated [8].

It is known that testing for epoxy allergy can be challenging and that epicutan tests may give false-negative results, thus testing with workplace substances is recommended [7, 13]. Unfortunately, our patient was not referred to the department of occupational and environmental medicine before he returned to work and continued his epoxy exposure. When further approaches were made to establish an association between exposure and his dermal reactions, he had already developed severe rhinitis. The patient then did not want to perform specific challenge testing because he feared worsening of symptoms. In Norway, patch tests with workplace substances can be performed in hospitals with a department of dermatology, nonetheless SiC-testing is limited to one specialist center only. Because attention has been drawn to possible adverse effects from the lower respiratory tract induced by SiC-testing [14], this limits the use of such testing further. In our case, the assessment of work relation thus had to rely on specialist assessment alone. Although questions that identify rhinitis symptoms at work, which improve on days away from work, may have a high sensitivity for detecting occupational disease, the specificity may be relatively low as shown for work-related asthma [15]. Thus, it is important to stress that exposure is reduced or avoided until assessment of possible work relation has been made.

Since specific IgE was not examined, and taking the limitations of the skin prick test into consideration, it is possible also that that the patient’s rhinitis was caused by another work related exposure. His work as an industrial flooring specialist may have involved mixed and multiple chemical exposures. For our patient, early recognition of an association between the dermal reactions and exposure probably could have secured the use of personal protective equipment or other means to stop or reduce exposure. Instead, the worker returned to exposure and developed severe work related upper airway symptoms. It is difficult to know if avoidance of exposure at an earlier stage could have prevented the development of respiratory symptoms for this worker. However, after the establishment of the diagnosis, the patient avoided further exposure to epoxy products and reported improvement of symptoms from the nose.

## Conclusions

We report an unusual case of multiple subcutaneous granulomas and severe work related rhinitis after a small injury with an epoxy-contaminated tool. It remains unclear whether the development of rhinitis in our patient may be a result of sensitization after intradermal deposition of epoxy or other irritants, or represents a delayed response to long-term airborne occupational exposure. However, the case highlights the challenges of establishing the association between exposure and dermal reactions. It may also be a reminder of the potential major role for the skin in respiratory allergy and that efforts should be made to prevent sensitization through skin exposure.

## Abbreviations

CRP, C-reactive protein; IgE, immunoglobulin-E; SiC, specific inhalation challenge test
